# Pharmacogenomic variation and chemotherapy-related toxicity profiles in pediatric patients with cancer in Tanzania: a cross-sectional study

**DOI:** 10.3389/fphar.2026.1886874

**Published:** 2026-07-20

**Authors:** Deogratias M. Katabalo, Baraka Fundo, Winfrida V. Minja, Heronima Joas Kashaigiri, Stanley Mwita, Anthony Cuthbert Liwa, Kristin Schroeder, Benson R. Kidenya

**Affiliations:** 1 Department of Pharmaceutics and Pharmacy Practice, School of Pharmacy, Catholic University of Health and Allied Sciences, Mwanza, Tanzania; 2 Department of Oncology, Bugando Medical Centre, Mwanza, Tanzania; 3 Department of Pharmacology, School of Medicine, Catholic University of Health and Allied Sciences, Mwanza, Tanzania; 4 Duke University Health Institute, Raleigh, NC, United States; 5 Department of Biochemistry and Molecular Biology, School of Medicine, Catholic University of Health and Allied Sciences, Mwanza, Tanzania

**Keywords:** chemotherapy-related toxicities, childhood cancers, drug-related adverse effects, genetic polymorphism, pharmacogenomics, Tanzania

## Abstract

**Background:**

Chemotherapy-related toxicities remain a major barrier to optimal outcomes in pediatric cancer care in low-resource settings. Genetic variation in drug-metabolizing and transport pathways contributes to interindividual differences in toxicity risk; however, pharmacogenomic data from African pediatric populations are limited. This study assessed the distribution of selected pharmacogenomic variants and chemotherapy-related toxicities profiles in Tanzanian children with cancer.

**Methods:**

A cross-sectional study was conducted among 155 pediatric patients with cancer (1–17 years) receiving chemotherapy at Bugando Medical Centre, Tanzania. Clinical data, clinician-reported toxicities, and patient-reported outcomes were collected. Eleven pharmacogenomic variants in genes involved in drug metabolism, transport, and detoxification pathways *(CYP3A5, SLC19A1, TPMT, NUDT15, GSTP1, NCF4, CYBA,* and *CEP72),* selected based on prior evidence of functional relevance and reported associations with chemotherapy response, were genotyped using the Agena MassARRAY® platform. Toxicities were graded using CTCAE version 5.0.

**Results:**

Hematologic toxicities, including anemia (50.3%) and leukopenia (37.4%), and mucocutaneous toxicities such as alopecia (38.1%) and oral ulcers (24.5%) were common. Reduced muscle strength was observed in 78.1% of patients. Reduced-function *CYP3A5* variants were frequent (CYP3A5*6: 38.1%; CYP3A5*3: 27.7%). Variants in *GSTP1* rs1695 (MAF 0.46), *CYBA* rs4673 (MAF 0.44), and *SLC19A1* rs1051296 (MAF 0.49) demonstrated substantial variability, indicating potential interindividual differences in drug metabolism and transport. However, no genotype–toxicity associations were assessed in this study.

**Conclusion:**

This study provides baseline data on pharmacogenomic variability among Tanzanian pediatric patients with cancer. The observed genetic diversity in pharmacogenes involved in drug metabolism and transport may be relevant to variability in chemotherapy-related toxicities, as reported in previous studies. These findings highlight the need for further research to evaluate genotype–toxicity relationships and to inform the future integration of pharmacogenomics into pediatric oncology care in resource-limited settings.

## Background

Pharmacogenomics is the study of the impact of genetic variations (or study of how genetic variations influence) on the efficacy and toxicity of pharmacological treatments (Wang, 2010). This field enhances our understanding of why patients may experience variable responses to the same dose of the same drug, even when administered for the same disease under comparable circumstances. These genetic distinctions can sometimes result in unintended consequences, such as diminished therapeutic effectiveness or the emergence of adverse drug reactions (ADRs) (Micaglio et al., 2021). In the context of pediatric cancer treatment, where chemotherapy is a fundamental component of care, genetic variations, particularly single-nucleotide polymorphisms (SNPs), significantly influence drug response and toxicity profiles (Palmirotta et al., 2018). The implementation of individualized or precision therapy within this framework has the potential to improve treatment outcomes by reducing the likelihood of severe ADRs, commonly identified as chemotherapy-related toxicities (Chopra et al., 2016).

Chemotherapy-related toxicities represent significant challenges that impact cure rates for children undergoing cancer treatment in Tanzania. Studies indicates that the 2-year survival rate for pediatric patients with cancer in Tanzania is between 20% to 40%, a contrast to the approximately 85% cure rate observed in high-income countries (Schroeder et al., 2017). Nonetheless, awareness of these issues is growing, and there is hope for improvement. The most commonly reported Chemotherapy-related toxicities among these children include nausea and vomiting, severe bone marrow suppression, peripheral neuropathy, diarrhea, mucosal ulcerations, and renal impairment (Moore and Groninger, 2013; Demilie et al., 2025). These toxicities may contribute to serious complications such as hypovolemic shock, severe bacterial and fungal infections secondary to immunosuppression, anemia, and bleeding disorders, while also negatively affecting nutritional status, physical functioning, treatment adherence, and quality of life (Chopra et al., 2016; Tay et al., 2022). By addressing the side effects of treatment, there is a pathway to enhance treatment adherence, paving the way for improved therapeutic success and a brighter future for pediatric oncology (Juthani et al., 2024).

To prevent or minimize the occurrence of Chemotherapy-related toxicities, patients are often prescribed supportive care medications in conjunction with chemotherapies for prophylaxis or treatment of specific adverse effects. For instance, antiemetics are used to prevent nausea and vomiting, co-trimoxazole is used to prevent bacterial infections, intravenous fluids are used for hydration to reduce the risk of kidney injury, colony-stimulating factors (e.g., filgrastim) are used to manage bone marrow suppression, and broad-spectrum antibiotics are used to treat severe infections (Katabalo et al., 2025a). Although these strategies are based on well-established clinical experience and evidence, the onset and severity of toxicities remain largely unpredictable across individual patients (Lee et al., 2023).

Clinicians therefore rely on their experience and observable patient factors, such as previous medication response, nutritional status, and established treatment guidelines, when individualizing therapy (Pedretti et al., 2023; Lee et al., 2023). However, evidence from high-income countries demonstrates that incorporating pharmacogenomic profiling into clinical decision-making significantly enhances treatment outcomes as reflected in established clinical guidelines from the Clinical Pharmacogenetics Implementation Consortium (CPIC) and the Dutch Pharmacogenetics Working Group (DPWG) for gene–drug pairs (Hippman and Nislow, 2019). By identifying specific SNPs associated with drug metabolism, transport, and sensitivity, clinicians can optimize chemotherapy dosing, monitor toxicity more closely, and select safer alternative agents, thereby reducing the incidence and severity of Chemotherapy-related toxicities (Palmirotta et al., 2018). Despite these benefits, the uptake and application of pharmacogenomic approaches in developing countries, including Tanzania, remain limited. In sub-Saharan Africa, data on genetic profiling among pediatric patients with cancer is scarce, largely due to low awareness among healthcare professionals and inadequate infrastructure for genomic and genetic testing (Katabalo et al., 2025b). Commonly studied SNP–chemotherapy pairings that inform individualized therapy include thiopurine S-methyltransferase (*TPMT*) and Nudix hydrolase 15 (*NUDT15*) with purine analogs (6-mercaptopurine and thioguanine); cytochrome P450 3A5 (*CYP3A5*) and centrosomal protein 72 (*CEP72*) with vincristine; glutathione S-transferase Pi 1 (*GSTP1*) with cisplatin; neutrophil cytosolic factor 4 (*NCF4*) with doxorubicin; and solute carrier family 19 member 1 (*SLC19A1*) with methotrexate (MTX). Individuals carrying defective variants in these genes are at increased risk of severe toxicities such as bone marrow suppression, neuropathy, nephrotoxicity, cardiotoxicity, and gastrointestinal complications (e.g., nausea, vomiting, mucosal ulceration, and loss of appetite) (Gerbek et al., 2018; Shalaby et al., 2024; Hurkmans et al., 2022).

Despite the growing evidence supporting pharmacogenomic-guided therapy in high-income settings, there is limited data on the distribution of clinically relevant pharmacogenomic variants among pediatric patients with cancer in sub-Saharan Africa. This lack of data limits the ability to contextualize toxicity risk and develop locally appropriate strategies for toxicity monitoring and management. Understanding the distribution of these variants and their potential relevance provides important baseline data for identifying candidate for pharmacogenomic targets and informing future studies aimed at evaluating their clinical utility in this population.

We hypothesized that pharmacogenomic variants involved in drug metabolism, transport, and detoxification are prevalent among Tanzanian pediatric patients with cancer and may be relevant to variability in observed toxicities during chemotherapy. Therefore, this study aimed to describe the distribution of selected pharmacogenomic variants and characterize toxicity profiles among pediatric patients with cancer in Tanzania, providing baseline data to inform future pharmacogenomic and clinical studies.

## Methods

### Study area

The study was conducted at the oncology department of the Bugando Medical Centre, the largest referral and university teaching hospital in the Lake Zone region, in the northwestern part of Tanzania. The hospital has the largest pediatric oncology unit (POU) in its cancer Centre, which is located in an area occupied by over 16 million people. Being the only hospital with cancer services in the region, it was an ideal area for data collection because all patients and their information are found at the cancer Centre.

### Study design

A cross-sectional study design was employed to describe the distribution of selected pharmacogenomic variants and to characterize toxicity profiles observed during chemotherapy. All eligible children undergoing chemotherapy or attending follow-up visits during the data collection period were enrolled in the study. Clinical information, clinician-observed toxicities, patient-reported outcomes, and blood samples for SNP genotyping were collected to determine the frequency of pharmacogenomic variants and to document the burden and pattern of toxicities observed during treatment. Toxicity data were collected to describe the clinical burden of treatment-related effects and to provide context for pharmacogenomic findings; no analyses were performed to assess associations between genetic variants and toxicities.

### Study population

All pediatric patients with confirmed cancer diagnosis, aged between 1 and 17 years, who were receiving chemotherapy or attending follow-up services after completing chemotherapy during the data collection period, were deemed eligible for inclusion in the study.

### Selection criteria

#### Inclusion criteria

This study included all children aged between 1 and 17 years with a histologically confirmed cancer diagnosis undergoing treatment with chemotherapy who had received at least one cycle (session) of chemotherapy. Bone marrow transplantation is not currently practiced at the study site; therefore, no participants with transplant history were included in this cohort.

#### Exclusion criteria

Children with severe anaemia requiring immediate clinical intervention or those undergoing blood transfusion at the time of data collection were excluded. These conditions may influence hematologic parameters and toxicity assessments, potentially confounding the evaluation of chemotherapy-related toxicities.

#### Sample size and sampling procedure

The minimum sample size for the study was determined using the Taro Yamane formula (1967): n = N/[1 + N (e^2^)] (Yamane, 1967). Where n is the required sample size, N is the estimated study population, and e is the margin of error. The study population was estimated at 250 children, based on the registered active pediatric patients with cancer receiving chemotherapy or attending follow-up at Bugando Medical Centre during the planning period. Using a 5% margin of error, the minimum required sample size was calculated as 154 participants and rounded to 155. As this study was designed as a descriptive cross-sectional study aimed at characterizing chemotherapy-related toxicities and the distribution of selected pharmacogenomic variants, the sample size calculation was based on the target clinical population rather than on allele frequency estimation or genotype–toxicity association analyses. A consecutive sampling approach was employed, whereby all eligible children attending the clinic during the data collection period were consecutively recruited until the desired sample size was achieved.

#### Study period and rationale for sampling

This study was conducted over 12-months period, from August 2024 to July 2025, and focused on children who received chemotherapy during this timeframe. Annually, approximately 250–300 pediatric patients with cancer are diagnosed and begin chemotherapy at the Pediatric Oncology Unit (POU) of Bugando Medical Centre. Prior to study initiation, a minimum sample size of 155 participants was calculated using the Taro Yamane formula based on the estimated annual patient population. Eligible participants were then recruited consecutively throughout the study period. Probability sampling was impractical due to unpredictable patient attendance and the need for timely processing of blood samples to ensure sample integrity prior to shipment to the laboratory in South Africa. Each participant was enrolled only once and assigned a unique study identification number; subsequent clinic or follow-up visits by the same participant were not considered new cases. By the end of the study period, the target sample size had been achieved.

## Data collection procedure

After obtaining ethical clearance, six research assistants were recruited and trained on study logistics and the use of data collection tools. The tools included: (i) a structured checklist for documenting objective clinical signs related to chemotherapy-related toxicities; (ii) the Pediatric PRO-CTCAE (Patient-Reported Outcomes version of the Common Terminology Criteria for Adverse Events) adopted from the National Cancer Institute (NCI); (Instrument and Form Builder, 2025); and (iii) a checklist for blood sample collection for DNA extraction and sequencing. All tools were pilot tested on 30 volunteers in three iterative rounds, after which necessary modifications were made to enhance clarity and usability.

Written informed consent was obtained from parents or guardians of all enrolled children while assent was obtained from children aged 7 years and above following an age-appropriate explanation of the study. Two research assistants were responsible for identifying eligible participants, explaining study procedures, and administering consent and assent process. Thereafter, two additional research assistants conducted structured assessments in a quiet, private room, including physical examinations, interviews with caregivers, and review of clinical files and laboratory records to identify documented chemotherapy-related toxicities. Toxicity assessments were conducted at the time of patient encounter during ongoing chemotherapy or follow-up visits, based on current clinical findings and patient-reported symptoms captured using the Ped-PRO-CTCAE with a 7 -day recall period. The study primarily captured acute and ongoing toxicities rather than cumulative or long-term effects. Neurological assessment, including evaluation of muscle strength, was performed as part of routine clinical examination based on standard pediatric physical assessment practices; no formal standardized neurological scoring tool was applied.

For each participant, the remaining two research assistants collected a 3 mL venous blood sample during routine clinical phlebotomy to minimize discomfort. Samples were collected in the standard blood-collection room, where participants were identified using the unique study numbers assigned during enrollment. Each tube was labeled with the participant’s study ID and hospital admission number, placed in a cool box, and transported to the Molecular Biology Laboratory at the Catholic University of Health and Allied Sciences (CUHAS). Samples were stored at −20 °C until DNA extraction. This process ensured accurate linkage of clinical data, PRO-CTCAE reports, and genotyping results while preventing duplication or misclassification.

## Laboratory analysis

### DNA extraction

Genomic DNA was extracted from whole-blood samples using the Zymo® DNA Extraction Mini Kit (Zymo Research), according to the manufacturer’s instructions. DNA concentration and purity were assessed using the Qubit 4 Fluorometer (Thermo Fisher Scientific) and agarose gel electrophoresis, respectively. Extracted DNA samples were stored at −20 °C until shipment to Inqaba Biotechnical Industries (South Africa) for genotyping analysis.

### SNP genotyping

Targeted genotyping was performed using the Agena Bioscience MassARRAY® System (Agena Bioscience), a MALDI-TOF–based platform for high-throughput SNP detection. Eleven SNPs were selected based on their reported involvement in drug metabolism, transport, and detoxification pathways, as well as prior evidence linking them to chemotherapy-related pharmacokinetics or toxicities in published studies ((Palmirotta et al., 2018; Bosch et al., 2008)). Selection was informed by pharmacogenomic databases, including PharmVar and ClinPGx (formerly PharmGKB), and supported by clinical annotations from CPIC where available. Priority was given to variants with established biological and clinical relevance rather than allele frequency considerations in specific populations. These variants were analyzed across the genes CYP3A5 (CYP3A5*3, rs776746; CYP3A5*6, rs10264272), SLC19A1 (rs1051296), TPMT (TPMT*2, rs1800462; TPMT*3B, rs1800460; TPMT*3C, rs1142345), NUDT15*3 (rs116855232), GSTP1 (rs1695), NCF4 (rs1883112), CYBA (rs4673), and *CEP72 (rs924607).* Genotyping assays were designed using the MassARRAY® Assay Design Suite and performed according to the manufacturer’s protocols. Genotype calls were assigned automatically using the MassARRAY® software and subsequently subjected to quality control procedures. Genotyping quality control was assessed using assay-level metrics generated by the platform across all plates. The overall genotyping call rate exceeded 96%, with minimal missing data across SNPs. Hardy–Weinberg equilibrium was evaluated for polymorphic variants, and no significant deviations were observed (p > 0.05), supporting the reliability of genotype calls. Samples with ambiguous or low-quality signal profiles were reanalyzed where necessary to ensure accurate genotype assignment. All 155 enrolled participants were successfully genotyped and included in the final analysis.

### Interpretation of MassARRAY data

Raw mass spectra generated from the MALDI-TOF analysis were imported into the MassARRAY® Insight software (Agena Bioscience). The software automatically compared the observed mass-to-charge (m/z) peaks with reference spectra in its internal database to identify allele-specific extension products. Genotype calls were assigned based on peak clustering and concordance with expected mass patterns. Relative peak intensities were used to confirm allele ratios and ensure call quality.

Allele definitions and reference sequences for all SNPs were retrieved from established genomic resources. Pharmacogene star alleles (e.g., CYP3A5*3, CYP3A5*6, TPMT*3C) were annotated using PharmVar and ClinPGx, whereas additional SNPs (*GSTP1, SLC19A1, NCF4, CYBA, CEP72*) were verified against NCBI dbSNP and Ensembl. Minor allele frequencies were compared with data from the 1000 Genomes Project (African/AFR population) to provide population-level context for the observed allele distributions; this dataset was not used for SNP selection. Clinical annotations for each variant were compiled from ClinPGx, the Clinical Pharmacogenetics Implementation Consortium (CPIC), PharmVar, and previously published peer-reviewed studies describing functional and toxicogenomic relevance.

### Data analysis procedure and statistical analysis

Data from the clinical checklists, Ped-PRO-CTCAE tool, and genotyping output were entered into Microsoft Excel, cleaned, coded, and subsequently exported to STATA version 15 for analysis. Continuous variables were summarized using means with standard deviations or medians with interquartile ranges, depending on distribution. Categorical variables were summarized as frequencies and percentages and presented in tables or figures.

Pharmacogenomic variant distributions were described using genotype frequencies, allele frequencies, and minor allele frequencies (MAF), calculated directly from the study cohort. Where applicable, star allele designations were derived from SNP-based genotypes in accordance with established pharmacogenomic nomenclatures. Chemotherapy-related toxicities were summarized according to clinician-observed findings and Ped-PRO-CTCAE grades across all organ/system domains. No inferential analyses or genotype–toxicity association tests were performed, as the study was designed to provide descriptive baseline data on pharmacogenomic variation and clinical toxicity profiles in this population. This study was reported in accordance with the Strengthening the Reporting of Observational Studies in Epidemiology (STROBE) guidelines and the Strengthening the Reporting of Genetic Association Studies (STREGA) recommendations where applicable.

## Results

### Demographics and clinical information of the participants

A total of 155 children aged 1–17 years were included in this study. The median age at diagnosis was 5 years (IQR: 3–13 years), with the largest age group consisting of children aged 1–4 years (41.9%). A male predominance was observed, accounting for 58.1% (n = 90) of participants. The majority of participants had no comorbidities (94.8%, n = 147), with only a small number exhibiting any comorbidity. The detailed distribution is presented in Table 1.

**TABLE 1:** Demographic characteristics and comorbidities.

Variable	Category	N (155)	%
Age group	1–4 years	65	41.9
	5–9 years	40	25.8
	10–14 years	22	14.2
	15–17 years	28	18.1
Gender	Male	90	58.1
	Female	65	41.9
Comorbidities	None	147	94.8
	Pneumonia	2	1.3
	Sickle cell disease	2	1.3
	Down’s syndrome	1	0.6
	Heart disease	1	0.6
	Tuberculosis	1	0.6
	Malnutrition	1	0.6

### Childhood cancer diagnosis among participants

A wide spectrum of childhood cancers was observed. The most frequent malignancy was nephroblastoma (Wilms tumour), accounting for 20.0% (n = 31) of all cases, followed by acute lymphoblastic leukemia (ALL) at 16.8% (n = 26) and retinoblastoma at 9.0% (n = 14). Several less common tumors, including Central Nervous System (CNS) tumors, germ cell tumors, Kaposi sarcoma, and other soft-tissue sarcomas, were also documented, although each represented ≤3% of the cases, as shown in Table 2.

**TABLE 2:** Distribution of childhood cancer diagnoses among participants (N = 155).

Tumor group	Diagnosis (subtype)	n	%
Leukemias	Acute lymphoblastic leukemia (ALL)	26	16.8
Leukemias	Acute myeloid leukemia (AML)	9	5.8
Lymphomas	Burkitt lymphoma	11	7.1
Lymphomas	Hodgkin lymphoma	7	4.5
Lymphomas	Non-Hodgkin lymphoma (NOS)	8	5.2
Lymphomas	Other lymphoma subtypes (ALCL, DLBCL)	5	3.2
Renal tumors	Nephroblastoma (Wilms tumor)	31	20.0
CNS tumors	Medulloblastoma, Pineoblastoma, Pilocytic astrocytoma, PNET	4	2.6
Soft tissue sarcomas	Embryonal rhabdomyosarcoma (ERMS)	8	5.2
Soft tissue sarcomas	Other soft tissue sarcomas (ARMS, NRSTS, Sarcoma NOS, Leiomyosarcoma)	4	2.6
Bone tumors	Osteosarcoma, Osteoblastoma, Chondrosarcoma	3	1.9
Neuroblastoma	Neuroblastoma	9	5.8
Retinoblastoma	Retinoblastoma	14	9.0
Germ cell tumors	Germ cell tumor	4	2.6
Other solid tumors	Hepatoblastoma, Kaposi sarcoma, NPC, Pulmonary blastoma, Sacrococcygeal tumor, SCC, Juvenile GCT	12	7.7

Abbreviations: ALL, acute lymphoblastic leukemia; AML, acute myeloid leukemia; ALCL, anaplastic large cell lymphoma; DLBCL, diffuse large B-cell lymphoma; ERMS, embryonal rhabdomyosarcoma; ARMS, alveolar rhabdomyosarcoma; NRSTS, non-rhabdomyosarcoma soft tissue sarcoma; NPC, nasopharyngeal carcinoma; GCT, germ cell tumour; NOS, not otherwise specified.

### Stage, risk, group, and grade classification of the observed childhood cancers diagnosis

The cancers diagnosed in this study were classified by stage, risk, group, or grade, depending on the tumor type. Among the tumors staged using the standard 1–4 staging system (n = 71), most children presented with advanced disease, with Stage 3 accounting for 46.5% (n = 33) and Stage 4 for 32.4% (n = 23). Early-stage presentations were less common, with only 2.8% in Stage 1% and 18.3% in Stage 2. For cancers classified by risk category, primarily leukemias (n = 61), the majority of patients were categorized as high risk (88.5%, n = 54), while only a small proportion fell into the standard-risk (6.6%) or low-risk (4.9%) groups. Retinoblastoma cases (n = 14) were grouped from C to E, with Group E being the most frequent category (71.4%, n = 10). Among tumors assessed histologically (n = 9), Grade 4 was the most common grade, representing 44.5% of those evaluated.

### Chemotherapy regimens prescribed

The recruited participants received a wide variety of chemotherapy regimens, reflecting the heterogeneity of pediatric cancer treatment protocols. In total, about 60 distinct regimen entries were recorded. However, many were the minor variations of a few backbone protocols. The most frequently used regimen was Vincristine + Carboplatin + Etoposide (9.7%, n = 15), followed by Actinomycin D + Vincristine + Doxorubicin (8.4%, n = 13), Cyclophosphamide + Etoposide (8.4%, n = 13), and Carboplatin + Etoposide + Cyclophosphamide + Doxorubicin (6.5%, n = 10). Each of the remaining regimens was prescribed to fewer than 5% of participants. To make the results more clinically interpretable, the heterogeneous free-text regimens were collapsed into clinically coherent backbones using a pre-specified hierarchy (AML induction with daunorubicin + cytarabine; asparaginase-containing; carboplatin + etoposide; cyclophosphamide + etoposide; vincristine-based; methotrexate/6-MP maintenance; other/targeted). This reflects common pediatric oncology protocols and groups agents with similar mechanisms and toxicity profiles. The distribution of regimens across these categories is presented in Table 3.

**TABLE 3:** Chemotherapy regimens prescribed among pediatric patients with cancer (N = 155).

Chemotherapy backbone	n	%
Vincristine-based (± Actinomycin, Cyclophosphamide, Doxorubicin, Methotrexate, etc.)	58	37.4
Carboplatin + Etoposide (± Cyclophosphamide, Doxorubicin, Vincristine, Bleomycin)	33	21.3
Cyclophosphamide + Etoposide (± Anthracycline, Platinum, Ifosfamide)	16	10.3
Methotrexate/6-Mercaptopurinemaintenance (± Vincristine, Dexamethasone)	10	6.5
AML induction (Daunorubicin + Cytarabine)	7	4.5
Asparaginase-containing regimens	7	4.5
Bleomycin/anthracycline/vinca combinations	6	3.9
Platinum doublets/triplets (Cisplatin, Carboplatin, Docetaxel, 5-Fluorouracil)	6	3.9
Other/ targeted (everolimus, arsenic trioxide, etc.)	12	7.7

### Clinician-observed signs, symptoms, and laboratory abnormalities

Chemotherapy-related toxicities were observed across multiple organ systems in this cohort. Overall, 148/155 participants (95.5%; 95% CI: 90.9–98.2) experienced at least one observed chemotherapy-related toxicity, while 100/155 participants (64.5%; 95% CI: 56.4–72.0) had two or more concurrent toxicities based on clinician-observed signs and symptoms. Hematologic toxicities were common: low hemoglobin levels were recorded in 78/155 participants (50.3%; 95% CI: 42.2–58.4; median 11.2 g/dL, IQR 9–12.8), and reduced red blood cell counts in 58/155 (37.4%; 95% CI: 29.8–45.5; median 4.37 × 10^12^/L, IQR 3.3–4.9). Clinically, pale skin was reported in 45/155 participants (29.0%), infectious dermatitis in 21/155 (13.6%), shortness of breath in 12/155 (7.7%), and bleeding in 9/155 (5.8%). Mucocutaneous toxicities were also frequent, including hair loss in 59/155 participants (38.1%; 95% CI: 30.4–46.2), mouth ulcers in 38/155 (24.5%; 95% CI: 18.0–32.1), and rashes in 32/155 (20.7%). Gastrointestinal involvement was primarily vomiting, reported in 21.9% of participants. Reduced muscle strength on clinical examination was noted in 121/155 participants (78.1%; 95% CI: 70.7–84.3). Renal toxicities were less frequent: 20.6% of participants experienced swelling, while laboratory indicators of renal dysfunction were rare, with only one child (0.65%) showing elevated creatinine and none exhibiting raised BUN levels. Hepatic abnormalities were similarly uncommon, with elevated liver enzymes observed in 7/155 participants (4.5%) and elevated bilirubin in 14/155 (9.0%). Table 4 summarizes clinician-observed signs, symptoms, and laboratory abnormalities recorded during routine clinical assessment and does not represent CTCAE-defined toxicity classifications. A full summary of observed toxicities is provided in Table 4. In addition, a descriptive summary of multiple observed toxicities according to diagnosis group and chemotherapy backbone is presented in Supplementary Table S1. Multiple observed toxicities (≥2 concurrent toxicities) were most frequent among children with acute lymphoblastic leukemia (84.6%), lymphoma (67.7%), acute myeloid leukemia (66.7%), and neuroblastoma (66.7%). Across treatment categories, multiple toxicities were most commonly observed among participants receiving asparaginase-containing regimens (100%), bleomycin/anthracycline/vinca combinations (83.3%), platinum doublets/triplets (83.3%), and vincristine-based regimens (69.0%). These findings are descriptive and should be interpreted cautiously given the relatively small sample sizes within several diagnostic and treatment subgroups.

**TABLE 4:** Summary of clinician-observed signs, symptoms, and laboratory abnormalities recorded during routine clinical assessment (N = 155).

System/organ	Feature observed	Present n (%)	Not present n (%)
Hematological	Pale skin	45 (29.0)	110 (71.0)
	Bleeding	9 (5.8)	146 (94.2)
	Low Hb (categorical)	78 (50.3)	77 (49.7)
	Low RBC count (categorical)	58 (37.4)	97 (62.6)
Respiratory	Shortness of breath	12 (7.7)	143 (92.3)
Gastrointestinal	Vomiting	34 (21.9)	121 (78.1)
Skin	Rash	32 (20.7)	123 (79.4)
	Hair loss	59 (38.1)	96 (61.9)
	Infectious dermatitis	21 (13.6)	134 (86.5)
Oral	Mouth ulcers	38 (24.5)	117 (75.5)
Neurological	Muscle weakness	121 (78.1)	34 (21.9)
Kidney	Swelling (any site)	32 (20.6)	123 (79.4)
	BUN raised	0 (0.0)	155 (100)
	Creatinine raised	1 (0.65)	154 (99.4)
Liver	Liver enzymes raised	7 (4.5)	148 (95.5)
	Bilirubin raised	14 (9.0)	141 (91.0)

### CTCAE-reported chemotherapy-related toxicities

Toxicities were reported across multiple organ systems, with varying frequency and severity. Oral mucositis affected 43.9% of children, most of whom experienced clinically significant impairment at grade 2 or higher (38.1%). Gastrointestinal toxicities were reported in 49.0% of participants, again dominated by grade 2 (39.4%) and grade 3 (9.7%) severity. Respiratory complaints were numerically most frequent (53.0%), but the majority were mild (grade 1, 24.5%), as were cutaneous changes, which were common (53.6%) yet largely grade 1 (43.9%). Cardiovascular events occurred in 32.9% of children, with nearly one-quarter (24.5%) presenting with moderate-to-severe (grade 2–3) findings. Neurological toxicities were less frequent (29.0%) but clinically important, as most were grade 2 (25.8%) with a few grade 3 (2.6%). Visual/perceptual symptoms were reported in 32.3% of cases, primarily grade 2 (16.1%), while attention/memory difficulties were less common (9.7%) yet almost all were grade 2 (9.0%). Pain was present in nearly half of the cohort (46.5%), although it was predominantly mild (grade 1, 33.6%). Sleep/wake disturbances affected 34.8%, with grade 1 predominance (25.8%). Mood alterations were identified in 28.4% of participants, again mostly mild (17.4% grade 1). Genitourinary toxicities were reported in 21.9%, with a significant proportion (16.1%) experiencing moderate-to-severe grades (Micaglio et al., 2021; Palmirotta et al., 2018). Detailed distributions are presented in Table 5.

**TABLE 5:** Chemotherapy-related toxicities by pediatric CTCAE domain among participants.

CTCAE domain	Grade 0 n (%)	Grade 1 n (%)	Grade 2 n (%)	Grade 3 n (%)	Grade 4 n (%)
Oral	87 (56.1)	9 (5.8)	50 (32.3)	9 (5.8)	–
Gastrointestinal	56 (36.1)	23 (14.8)	61 (39.4)	15 (9.7)	–
Respiratory	73 (47.1)	38 (24.5)	33 (21.3)	11 (7.1)	–
Cardiovascular	104 (67.1)	13 (8.4)	29 (18.7)	9 (5.8)	–
Cutaneous	72 (46.5)	68 (43.9)	12 (7.7)	3 (1.9)	–
Neurological	110 (71.0)	1 (0.6)	40 (25.8)	4 (2.6)	–
Visual/Perceptual	105 (67.7)	17 (11.0)	25 (16.1)	8 (5.2)	–
Attention/Memory	140 (90.3)	–	14 (9.0)	1 (0.6)	–
Pain	83 (53.6)	52 (33.6)	9 (5.8)	11 (7.1)	–
Sleep/Wake	101 (65.2)	40 (25.8)	2 (1.3)	12 (7.7)	–
Mood	111 (71.6)	27 (17.4)	10 (6.5)	5 (3.2)	2 (1.3)
Genitourinary	121 (78.1)	9 (5.8)	16 (10.3)	9 (5.8)	–

### Distribution of pharmacogenomic variants among participants

We evaluated the distribution of 11 pharmacogenomic variants in all recruited participants. Three SNPs, TPMT*2 (rs1800462), TPMT*3B (rs1800460), and NUDT15*3 (rs116855232), were monomorphic, with all participants carrying the homozygous reference CC genotype. In contrast, TPMT*3C (rs1142345) was present in 16.1% of patients (MAF 0.08). In addition to TPMT, variants in other drug-metabolizing genes were also observed. CYP3A5*3 (rs776746) was present in 27.1% of participants carrying the reduced-function C allele (MAF 0.14), while CYP3A5*6 (rs10264272) showed a higher prevalence (38.1% carriers, MAF 0.21). CEP72 (rs924607), previously reported in relation to vincristine toxicity, was less frequent (11.0% CT, MAF 0.06). Variants in detoxification pathways were also common. GSTP1 (rs1695) was highly polymorphic (MAF 0.46), with 72.9% of participants carrying at least 1 G allele. Similarly, oxidative stress–related variants were observed, with CYBA (rs4673) showing 63.9% carriers of the A allele (MAF 0.44) and NCF4 (rs1883112) showing 23.2% carriers of the A allele (MAF 0.12). SLC19A1 (rs1051296), a folate transporter gene, showed a near-equal allele distribution (MAF 0.49), indicating substantial interindividual variability within the cohort. Overall, the study population demonstrated a heterogeneous distribution of pharmacogenomic variants across genes involved in drug metabolism, transport, and detoxification (Table 6). These variant frequencies represent the overall distribution within the study population and were not analyzed in relation to specific chemotherapy exposures or toxicity outcomes.

**TABLE 6:** Distribution of pharmacogenomic variants in the study population (descriptive analysis).

Gene (SNP)	Genotype distribution (n, %)	Minor allele frequency, MAF (95% CI)	% Carriers of risk allele	Reported functional relevance (based on prior literature)
CYP3A5*6 (rs10264272)	CC: 96 (61.9%), CT: 52 (33.6%), TT: 7 (4.5%)	0.21 (T; 0.17–0.26)	38.1% (CT + TT)	Reduced CYP3A5 activity; has been associated with altered vincristine metabolism in previous studies (Bosilkovska et al., 2016)
*SLC19A1* (rs1051296)	AA: 36 (23.2%), AC: 81 (52.3%), CC: 38 (24.5%)	0.49 (A; 0.44–0.55)	75.5% (AC + AA)	Variants in SLC19A1 have been reported to influence methotrexate pharmacokinetics in prior studies, particularly among homozygous variant genotypes (Lima et al., 2014)
TPMT*3C (rs1142345)	CC: 1 (0.6%), CT: 24 (15.5%), TT: 130 (83.9%)	0.08 (C; 0.06–0.12)	16.1% (CT + CC)	Has been associated with thiopurine-related toxicity in prior studies (Jantararoungtong et al., 2021)
NUDT15*3 (rs116855232)	CC: 155 (100%)	No variant allele detected	0%	No NUDT15*3 variant detected in this cohort; however, other untested variants may still influence thiopurine response (Yin et al., 2017)
*GSTP1* (rs1695)	AA: 42 (27.1%), AG: 84 (54.2%), GG: 29 (18.7%)	0.46 (G; 0.40–0.52)	72.9% (AG + GG)	Has been associated with platinum and alkylating-agent toxicity in previous studies (Kim et al., 2022)
TPMT*3B (rs1800460)	CC: 155 (100%)	No variant allele detected	0%	No TPMT*3B variant detected; other TPMT variants may still influence thiopurine response (Ataseven et al., 2022)
TPMT*2 (rs1800462)	CC: 155 (100%)	No variant allele detected	0%	No TPMT*2 variant detected; other TPMT variants may still influence thiopurine response (Ataseven et al., 2022)
*NCF4* (rs1883112)	AA: 2 (1.3%), AG: 34 (21.9%), GG: 119 (76.8%)	0.12 (A; 0.09–0.16)	23.2% (AG + AA)	Has been associated with etoposide- and anthracycline-related toxicities in prior studies (Cascales et al., 2013)
*CYBA* (rs4673)	AA: 36 (23.2%), AG: 63 (40.7%), GG: 56 (36.1%)	0.44 (A; 0.38–0.49)	63.9% (AA + AG)	Has been associated with oxidative stress–related toxicity, including anthracycline effects, in prior studies (Yang et al., 2021)
CYP3A5*3 (rs776746)	CC: 1 (0.6%), CT: 42 (27.1%), TT: 112 (72.3%)	0.14 (C; 0.11–0.19)	27.7% (CC + CT)	Reduced CYP3A5 activity; has been associated with vincristine-related toxicity in previous studies (Bosilkovska et al., 2016)
*CEP72* (rs924607)	CC: 138 (89.0%), CT: 17 (11.0%)	0.06 (T; 0.03–0.09)	11.0% (CT)	Exploratory associations with vincristine-induced neuropathy have been reported in prior studies (Christofyllakis et al., 2024)

MAF calculated from observed genotype distribution in the study cohort. Clinical relevance statements are based on previously published pharmacogenomic studies and guideline databases and do not reflect associations evaluated within the current study.

## Discussion

The extent and severity of chemotherapy-related toxicities are influenced by multiple factors, highlighting the importance of comprehensive evaluation of contributing elements for effective clinical management (Tola et al., 2023). In the current study, we described both observed and reported toxicities, alongside the distribution of selected pharmacogenomic variants among Tanzanian children with cancer. This study was not designed to assess statistical associations between genetic variants and toxicities; therefore, the findings should be interpreted as descriptive and hypothesis-generating. A total of 155 participants were enrolled, a sample size comparable to other pediatric oncology studies conducted in Tanzania. Given Bugando Medical Centre’s (BMC) average annual caseload of approximately 300 new pediatric cancer cases and a 2-year survival rate of around 40% (Schroeder et al., 2017), the 1-year cross-sectional design provides a reasonable representation of the patient population. The demographic characteristics observed, including a male predominance (58.1%), a median age of 5 years, and a higher proportion of children under 4 years of age, are consistent with previous studies conducted at BMC and other pediatric oncology units in northern Tanzania (Schroeder et al., 2017; Majaliwa et al., 2023). These findings suggest a relatively stable demographic pattern among pediatric patients with cancer in this setting. In contrast, a study from South Africa reported differing demographic distributions, which may reflect methodological differences, including the use of retrospective data, potentially introducing sampling bias (Otoo et al., 2020).

The diagnostic distribution of childhood cancers identified in this study is consistent with patterns reported at Muhimbili National Hospital (MNH), another major pediatric oncology center in Tanzania, where nephroblastoma and acute lymphoblastic leukemia (ALL) are among the most frequently diagnosed cancers (Efraim et al., 2022). These conditions typically peak between the ages of two and 6 years, which corresponds with the age distribution observed in our cohort. In the Lake Zone, where this study was conducted, a higher prevalence of congenital anomalies such as aniridia and genitourinary malformations, recognized risk factors for nephroblastoma, has been reported, which may partly explain this observation (Mashuda et al., 2014). Additionally, institutional cancer registry data from BMC indicate a gradual increase in nephroblastoma cases since 2017, contributing to its current predominance over Burkitt lymphoma. In contrast, studies from other regions of northern Tanzania have reported a higher prevalence of leukemias and lymphomas, which may reflect regional differences in genetic factors, environmental exposures, or access to healthcare services (Schroeder et al., 2017; Majaliwa et al., 2023).

Most participants presented with advanced disease, with the majority of staged cancers classified as stage III or IV, and high-risk or high-grade tumors predominating. These findings are consistent with observations from the Kilimanjaro Christian Medical Centre (KCMC) Pediatric Oncology Unit (Majaliwa et al., 2023) but differ from earlier reports at BMC that included a higher proportion of outpatient cases and therefore fewer severe presentations (Katabalo et al., 2025a). Late presentation remains a significant challenge in Tanzanian pediatric oncology and is associated with poorer clinical outcomes (Sanga et al., 2022). Contributing factors may include delayed referrals from peripheral health facilities, limited diagnostic capacity, and the complexity of Tanzania’s multi-tiered referral system, all of which can prolong the time to definitive diagnosis and initiation of treatment (Mai et al., 2020).

All participants received chemotherapy according to institutionally adapted protocols. Most were treated with vincristine-based regimens, which are generally associated with a lower toxicity burden compared with anthracycline- or platinum-containing combinations. This may partly account for the predominance of mild or less severe toxicities observed in both clinician assessments and patient-reported Ped-PRO-CTCAE data. The regimens used are consistent with existing Tanzanian pediatric oncology guidelines and those employed at centers in northern and eastern Tanzania (National-Cancer-Treatment-Guidelines, 2025; Shimizu et al., 2025). Treatment decisions in this context are influenced not only by disease type and stage but also by drug availability, access, and clinician discretion. Most of the drugs used are included in the Tanzanian Essential Medicines List, with the exception of a few specialized agents, such as arsenic trioxide, which were used in a limited number of patients (Petricca et al., 2023). Descriptive subgroup analyses suggested variation in the burden of multiple observed toxicities across diagnostic groups and chemotherapy backbones; however, the study was not designed or powered to evaluate diagnosis-specific or regimen-specific toxicity associations, and these findings should therefore be interpreted cautiously.

Chemotherapy-related toxicities were observed across multiple organ systems, with hematologic and mucocutaneous effects being the most common. The high prevalence of anemia and leukopenia is consistent with the known myelosuppressive effects of combination chemotherapy and aligns with findings from other sub-Saharan African studies conducted in Uganda (Mutyaba et al., 2019). Mucocutaneous manifestations such as alopecia, mucositis, and dermatitis were also frequent, consistent with previous reports of epithelial dose-limiting toxicities in pediatric patients (Naveed et al., 2019). Reduced muscle strength was observed in a large proportion of participants (78.1%). Although peripheral neuropathy has been reported among pediatric cancer patients receiving chemotherapy in previous studies, with frequencies ranging from 70% to 80% (Mora et al., 2016), reduced muscle strength assessed through routine clinical examination is not equivalent to peripheral neuropathy and should therefore be interpreted with caution. Reduced muscle strength may also reflect underlying disease burden, malnutrition, general physical deconditioning, and the effects of combination chemotherapy regimens and concomitant therapies, which were not specifically controlled for in this study. In contrast, renal and hepatic toxicities were infrequent, which may be related to factors such as dose adjustments, hydration practices, and supportive care measures.

Furthermore, given the limited pharmacogenomic data available for Tanzanian pediatric oncology populations, this study provides baseline information on the distribution of selected variants in genes involved in drug metabolism, transport, and detoxification. Loss-of-function alleles in *CYP3A5* (*3 and *6) were observed at notable frequencies, consistent with reports from African and Asian populations (Mlakar et al., 2016). CYP3A5 is among the most frequently investigated pharmacogenes in relation to vincristine response and toxicity in pediatric oncology populations (Egbelakin et al., 2011). In contrast, the *CEP72* (rs924607) variant, which has been reported in relation to vincristine-induced neuropathy, was relatively infrequent in this cohort. This finding is consistent with reports by Diouf et al., who observed lower frequencies of the variant (T) allele among individuals of African ancestry compared with those of European ancestry (Diouf et al., 2015). Although both *CYP3A5* and *CEP72* variants have been implicated in vincristine-related toxicity in prior studies, genotype–toxicity associations were not evaluated in the present study; therefore, the clinical significance of these variants within this cohort remains uncertain.

*SLC19A1* (rs1051296), a gene encoding a folate transporter involved in methotrexate uptake, showed a near-equal allele distribution (MAF 0.49), indicating substantial interindividual variability within the study population. Previous studies have reported inconsistent findings regarding the association between this variant and methotrexate-related toxicities, with results influenced by factors such as age group, cancer type, dosing regimens, and study design (Wang et al., 2014; Gutierrez-Camino et al., 2014). Although prior studies have suggested that SLC19A1 variants may influence methotrexate transport and toxicity profiles, such relationships were not evaluated in the present study and therefore cannot be inferred from the current findings (Hernandez et al., 2024; Karpa et al., 2024).

Variants in *GSTP1* (rs1695), *CYBA* (rs4673), and *NCF4* (rs1883112) were observed at notable frequencies in this cohort. These genes are involved in oxidative stress and detoxification pathways and have been investigated in relation to chemotherapy-related toxicities in previous studies. The high prevalence of the *GSTP1* G allele (MAF 0.46) is consistent with findings from other populations and has been associated with reduced glutathione S-transferase activity in prior studies (Kim et al., 2022). However, evidence regarding its clinical relevance has been inconsistent, and some reported associations did not remain significant after correction for multiple testing. Similarly, CYBA and NCF4 variants have been investigated in relation to anthracycline- and etoposide-related toxicities, with findings varying across populations and study designs. Previous studies have also suggested that associations involving NCF4 may be genotype-specific, with significant effects reported primarily among homozygous variant carriers rather than carriers in general (Bansal et al., 2020). As genotype–toxicity associations were not evaluated in the present study, the clinical significance of these variants within this cohort remains uncertain.

TPMT and NUDT15 are among the most extensively studied pharmacogenes in pediatric oncology and have been incorporated into international pharmacogenomic guidelines for thiopurine dosing because of their established association with treatment-related toxicity. Consequently, characterization of their distribution in underrepresented populations remains important for future implementation efforts. Furthermore, *TPMT* and NUDT15*3 variants were predominantly monomorphic in this cohort, except for TPMT*3C (rs1142345), which was present in 16.1% of participants. This frequency is broadly consistent with the 9.8% reported among Zimbabwean children by Chikondowa et al., reflecting known patterns of interpopulation variability across sub-Saharan Africa, where allele frequencies may differ modestly due to distinct ancestral and demographic factors (Chikondowa et al., 2023; Relling et al., 2011; Moriyama et al., 2016; Relling et al., 2019).

Although this study did not evaluate statistical associations between pharmacogenomic variants and chemotherapy-related toxicities, the pharmacogenes investigated have previously been implicated in drug metabolism, transport, and oxidative stress pathways relevant to chemotherapy response. The observed variant frequencies provide baseline pharmacogenomic data for an underrepresented population and support the need for future adequately powered studies to evaluate genotype–toxicity relationships in African pediatric oncology settings.

### Study limitations

The relatively small sample size limited statistical power, particularly for drug- and toxicity-specific subgroup analyses and precluded formal evaluation of genotype–toxicity associations. In addition, the sample size was determined using a population-based approach for a descriptive cross-sectional study and was not based on allele-frequency precision calculations. Consequently, the precision of frequency estimates for rare genetic variants may have been limited. The use of a cross-sectional design and partial reliance on clinical documentation may have led to underestimation or misclassification of certain toxicities and limited the ability to establish temporal relationships or causality. Furthermore, no formal adverse event causality assessment tool was applied; therefore, observed toxicities cannot be definitively attributed to chemotherapy alone and may also reflect the underlying malignancy, concomitant therapies, or other clinical factors. In addition, exclusion of children with severe anemia requiring immediate clinical intervention or blood transfusion may have resulted in underestimation of the true prevalence and severity of high-grade hematologic toxicities in the broader pediatric oncology population. The use of a hospital-based consecutive sampling approach may limit the generalizability of the findings, and the study population may not fully represent the broader pediatric oncology population in Tanzania, particularly with respect to disease stage, healthcare access, and follow-up patterns. Specifically, as participants were recruited from a single tertiary care center, the study population may have included a higher proportion of patients with advanced disease, those able to access specialized care, or those retained in follow-up, compared to the broader pediatric cancer population. The analysis was restricted to a selected panel of SNPs and did not include comprehensive coverage of all relevant pharmacogenes or functional variants. Therefore, the absence of certain variants, such as NUDT15*3, TPMT*2, or TPMT*3B alleles, does not exclude the presence of other untested variants that may influence drug response. Moreover, the study did not collect data on ancestry, ethnicity, region of origin, or population substructure within the cohort. Given the substantial genetic diversity across African populations, the observed allele frequencies may not be fully representative of all Tanzanian populations. Finally, the heterogeneity of chemotherapy regimens and supportive care practices may have influenced the observed toxicity profiles. Despite these limitations, the study provides important baseline data on pharmacogenomic variation in an underrepresented population and highlights areas for future research to support the development of pharmacogenomics-informed cancer care in low-resource settings.

## Conclusion

In this cohort of Tanzanian children with cancer, toxicities occurring during chemotherapy treatment were common, with hematologic abnormalities, mucocutaneous manifestations, and muscle weakness among the most frequently observed findings. The study also identified notable variability in selected pharmacogenes, including *CYP3A5, GSTP1, CYBA, NCF4, TPMT,* and *SLC19A1*. These findings provide baseline data on pharmacogenomic variation in pediatric patients with cancer in Tanzania. However, as this study did not assess genotype–toxicity associations, the clinical implications of these variants cannot be directly inferred from the current findings. Future large, well-designed, multi-center studies are needed to evaluate genotype–toxicity relationships and to inform the potential integration of pharmacogenomics into pediatric oncology care in low-resource settings.

## Data Availability

The datasets presented in this study can be found in online repositories. The names of the repository/repositories and accession number(s) can be found below: https://ngdc.cncb.ac.cn/gvm/, GVM001302.
