# Supplementary material for: Pharmacogenomic variation and chemotherapy-related toxicity profiles in pediatric patients with cancer in Tanzania: a cross-sectional study
Source: Front Pharmacol. 2026 Jul 20;17:1886874. doi: 10.3389/fphar.2026.1886874 (PMC13429678; doi:10.3389/fphar.2026.1886874)
Supplement: Supplementary file 1 [file Table1.docx]

**Supplementary Table S1. Distribution of participants with multiple observed toxicities according to diagnosis group and chemotherapy backbone**

| **A. Diagnosis group and multiple observed toxicities** | | |
| --- | --- | --- |
| **Diagnosis group** | **Total (n)** | **≥2 observed toxicities n (%)** |
| Acute lymphoblastic leukemia (ALL) | 26 | 22 (84.6) |
| Acute myeloid leukemia (AML) | 9 | 6 (66.7) |
| Lymphoma | 31 | 21 (67.7) |
| Nephroblastoma | 31 | 19 (61.3) |
| Neuroblastoma | 9 | 6 (66.7) |
| Other solid tumors | 27 | 17 (63.0) |
| Retinoblastoma | 14 | 6 (42.9) |
| Rhabdomyosarcoma | 8 | 3 (37.5) |
| Total | 155 | 100 (64.5) |
| **B. Chemotherapy backbone and multiple observed toxicities** | | |
| **Chemotherapy backbone** | **Total (n)** | **≥2 observed toxicities n (%)** |
| Vincristine-based (± Actinomycin, Cyclophosphamide, Doxorubicin, Methotrexate, etc.) | 58 | 40 (69.0) |
| Carboplatin + Etoposide (± Cyclophosphamide, Doxorubicin, Vincristine, Bleomycin) | 33 | 17 (51.5) |
| Cyclophosphamide + Etoposide (± Anthracycline, Platinum, Ifosfamide) | 16 | 9 (56.3) |
| Methotrexate/6-Mercaptopurine maintenance (± Vincristine, Dexamethasone) | 10 | 5 (50.0) |
| AML induction (Daunorubicin + Cytarabine) | 7 | 5 (71.4) |
| Asparaginase-containing regimens | 7 | 7 (100.0) |
| Bleomycin/anthracycline/vinca combinations | 6 | 5 (83.3) |
| Platinum doublets/triplets (Cisplatin, Carboplatin, Docetaxel, 5-Fluorouracil) | 6 | 5 (83.3) |
| Other / targeted (everolimus, arsenic trioxide, etc.) | 12 | 7 (58.3) |
| Total | 155 | 100 (64.5) |

*Note: Multiple observed toxicities were defined as the presence of two or more clinician-observed chemotherapy-related toxicities during the study period. Results are presented descriptively without inferential statistical comparisons.*
